# The indirect association of job strain with long-term sickness absence through bullying: a mediation analysis using structural equation modeling

**DOI:** 10.1186/s12889-016-3522-y

**Published:** 2016-08-22

**Authors:** Heidi Janssens, Lutgart Braeckman, Bart De Clercq, Annalisa Casini, Dirk De Bacquer, France Kittel, Els Clays

**Affiliations:** 1 Department of Public Health, Ghent University, University Hospital, block 4k3, De Pintelaan 185, B 9000 Ghent, Belgium; 2 Research Centre Social approaches of Health, School of Public Health, Université libre de Bruxelles, B-1070 Bruxelles, Belgium

**Keywords:** Absenteeism, Bullying, Structural equation modeling

## Abstract

**Background:**

In this longitudinal study the complex interplay between both job strain and bullying in relation to sickness absence was investigated. Following the “work environment hypothesis”, which establishes several work characteristics as antecedents of bullying, we assumed that job strain, conceptualized by the Job-Demand-Control model, has an indirect relation with long-term sickness absence through bullying.

**Methods:**

The sample consisted of 2983 Belgian workers, aged 30 to 55 years, who participated in the Belstress III study. They completed a survey, including the Job Content Questionnaire and a bullying inventory, at baseline. Their sickness absence figures were registered during 1 year follow-up. Long-term sickness absence was defined as at least 15 consecutive days. A mediation analysis, using structural equation modeling, was performed to examine the indirect association of job strain through bullying with long-term sickness absence. The full structural model was adjusted for several possible confounders: age, gender, occupational group, educational level, company, smoking habits, alcohol use, body mass index, self-rated health, baseline long-term sickness absence and neuroticism.

**Results:**

The results support the hypothesis: a significant indirect association of job strain with long-term sickness absence through bullying was observed, suggesting that bullying is an intermediate variable between job strain and long-term sickness absence. No evidence for the reversed pathway of an indirect association of bullying through job strain was found.

**Conclusions:**

Bullying was observed as a mediating variable in the relation between job strain and sickness absence. The results suggest that exposure to job strain may create circumstances in which a worker risks to become a target of bullying. Our findings are generally in line with the work environment hypothesis, which emphasizes the importance of organizational work factors in the origin of bullying.

This study highlights that remodeling jobs to reduce job strain may be important in the prevention of bullying and subsequent sickness absence.

## Background

Management of sickness absence remains one of the major concerns of most employers and governments, in order to reduce the related costs for companies and society. In Belgium, the levels of sickness absence and associated costs have increased during last decade. This finding is mainly attributed to long spells of sick leave [1]. In 2010, the total burden of sickness absence, for an employer with 200 workers, was estimated at €1.053.360, which includes both direct (secured wages) and indirect costs (reorganizational problems, replacement costs, quality loss, reduced productivity) [1].

Therefore, employers put emphasis on repressive measures which focus on sickness absence control. But also more preventive strategies toward sickness absence, which concentrate on redesign of jobs to improve several jobs characteristics, have gained growing attention.

### Job strain and sickness absence

Several work-related psychosocial stressors are considered as risk factors for sickness absence.

The Job-Demand-Control (JDC)-model of Karasek [2] is one of the most leading job stress models since 1980′s and assumes that the combination of high demands and low control (job strain) will result in stress reactions, such as high blood pressure [3, 4] and decreased psychological well-being [5]. Besides these health and well-being outcomes, several authors also demonstrated a relation between job strain and future sickness absence [6, 7].

Although numerous studies have demonstrated the association between several work stressors and health variables, the processes leading to these health problems are less investigated and this research mainly focuses on the individual physiological changes. However, exposure to psychosocial work stressors not only causes physiological changes at the individual level, but can also have effects on the social relations between colleagues. One of the more extreme forms of dysfunctional social interaction between workers is interpersonal conflict which possibly escalates in workplace bullying [8].

### Bullying and sickness absence

The phenomenon of workplace bullying refers to the prolonged and repeated exposure to frequent aggressive and hostile behaviors at work, such as excessive criticism, withholding necessary information, spreading of rumors and social isolation [9].

Although a generally accepted definition of bullying is lacking in literature, there are some consistencies between the most commonly used definitions. There is agreement that bullying consists of repeated negative acts towards one or more victims [9–12]. Some definitions explicitly mention the persistent character of the bullying behavior [11, 12] or underline that the victim perceives difficulties to defend him or herself [9] and so point at the imbalance of power. As proposed by Notelaers [13], bullying essentially is a process, frequently triggered by a work-related conflict [14] in which the victim becomes increasingly targeted and demonstrates an inability to cope with the whole situation [15].

While classical psychosocial work stressors (such as job demands, control, support) have frequently been studied, the impact of being a victim of bullying on individual health, well-being and sickness absence is less investigated. Nevertheless, bullying is reported to be a serious problem, with possibly severe consequences for health and well-being of the individual worker. Being a target of bullying has been associated with psychological problems [16–18], but also with physical illness [19].

Only a few authors [20–23] demonstrated that bullying prospectively increased the risk for sickness absence, however these studies were mainly restricted to samples consisting of healthcare workers. Another notable finding is that, when investigating several psychosocial risk factors, bullying seems to have the strongest association with sickness absence [24]. In line with these findings, we assume:

*Hypothesis 1:* High levels of bullying are positively associated with long-term sickness absence during follow-up.

### Interplay between job strain and bullying

Both organizational and individual factors have been described as potential antecedents of workplace bullying. Individual factors related to being a victim of bullying are shyness [25], neuroticism [26] and low social skills [27]. Workplace and organizational factors that are demonstrated to be associated with bullying at work are diverse: high workload [11, 28], low work control [25, 28, 29], role conflicts [25], role ambiguity [25], change at work [30], job insecurity [30], poor organizational [31, 32] and social climate [25] and destructive leadership types (such as laissez-faire leadership and aggressive leadership) [33, 34]. Most studies investigating the determinants of bullying are based on the “work environment hypothesis”, which essentially assumes that workplace bullying can be attributed to a stressful work environment [11, 28]. A general remark on the majority of the literature is that the link with an explanatory framework is lacking. Until now, only a few authors applied existing job stress models in order to explain the origin of bullying. The JDC-model was tested in relation to workplace bullying and these studies supported the strain hypothesis indicating that high job strain (which is the combination of high demands and low control) leads to reports of bullying [29, 35–37].

In a qualitative study, examining the antecedents of workplace bulling, Ballien proposed a three-way-model explaining how job stress possibly creates a matrix for workplace bullying [38]. One of the pathways, explaining the link between job stress and bullying is based on the frustration-aggression hypothesis [39]. A worker who experiences “frustration”, because of job strain, may react with an inefficient coping behavior (eg. persistent complaining about the situation and distancing from work with decreased performance). This behavior possibly results in confronting the existing habits in the workplace or in violating expectations, which in turn leads to reactive behavior of the co-workers. In this manner, the worker, experiencing job strain, puts him- or herself into a risk-full situation for victimization by others. Yet, it is also possible that a worker confronted with work-related stressors, has less energy and strength and becomes exhausted. These reduced workers’ resources can imply that they become “easy targets”, who offer little resistance against workplace bullying [40]. In line with this framework, we assume:

*Hypothesis 2:* High levels of job strain are positively associated with high levels of bullying.

In order to disentangle the intermediate steps from job stress to the occurrence of health problems and related sickness absence, the complex interplay between both stressors (job strain and bullying) in relation to sickness absence will be examined. As far as we know, only one author has combined both stressors in order to explain the relation between job strain and a health outcome, revealing that workplace bullying mediated the relation between job strain and depression/sleep disturbances [41]. However, several methodological shortcomings can be mentioned on this study. The results are established on cross-sectional findings, which hamper the possibility to draw conclusions with respect to causality. Second, all results are based on self-reports, which produces a problem of common method bias. Third, the applied mediation analysis, based on the procedure proposed by Baron and Kenny, has several limitations [42].

**Fig. 1:**
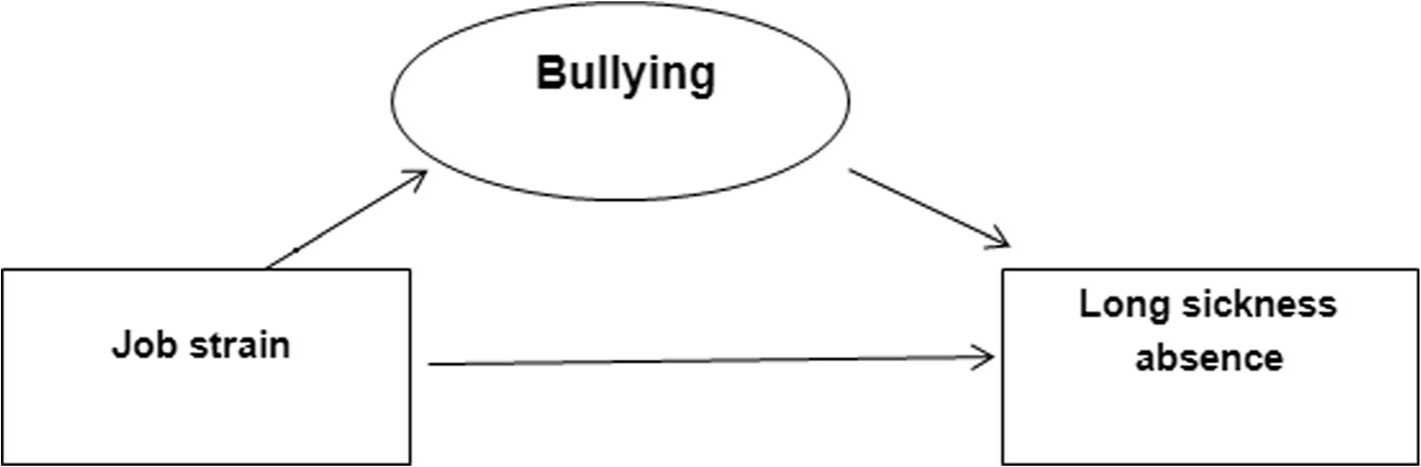
Hypothesized model

The present study wants to overcome these limitations and get insight into the processes contributing to the health harming effect of job strain, by using long-term sickness absence as an objective outcome measure, based on registered data in a longitudinal design. Furthermore, the application of new statistical methods, gives us the opportunity to get deeper knowledge on the relationship between several stressors and their consequences on ill-health. This paper wants to integrate the JDC-model and bullying concepts in relation to long-term sickness absence (LTSA), in order to get more insight into the complex interplay between job strain and bullying. Based on the previous findings of Baillien and Takaki [31, 33], we hypothesize that job strain will be indirectly associated with LTSA through workplace bullying. Job strain, which is the combination of high job demands and low control, possibly creates a work atmosphere in which bullying behavior will escalate, in turn causing sickness absence. We hypothesize that at least a part of LTSA caused by job strain, can be explained by being a target of bullying (Fig. 1). The aim of this study was to test a mediation model in which the indirect relation of job strain to LTSA through bullying was estimated, using structural equation modeling.

*Hypothesis 3:* Job strain is indirectly associated with LTSA through bullying

## Methods

### Study sample and procedure

The Belstress III study, conducted in 2004 in seven Belgian companies (comprising public administration, health care and social work sectors and manufacturing company), was a follow-up study aiming to identify risk factors for sick leave at work [43].

The workers, aged 30 to 55 years, were invited to participate in the study. The response rate was 30.4 %, representing a total of 2983 participants, and was lower in the lower occupational groups. Written informed consent was obtained from all participants. Analysis of the non-respondent characteristics revealed no difference with respect to gender or age. The study sample consisted of 1372 men and 1611 women, and the majority (72 %) was employed full-time. At baseline, all participants completed a self-administered questionnaire including standardized measures for individual and socio-demographic variables, health behaviors and characteristics of the psychosocial work environment. Thereafter, objective sickness absence data were collected prospectively during 12 months follow-up, starting from the day on which the questionnaire was filled out. The Belstress III study was approved by the ethics committees of the Ghent University Hospital and the Faculty of Medicine of the Université libre de Bruxelles.

### Measures

#### Job strain

Job strain was operationalized, using the recommended scales “job demands” and “decision latitude” of the Job Content Questionnaire (JCQ) [2]. The JCQ is based on the JDC-model and is one of the most widely used instruments to assess the psychosocial work environment. Job demands were measured using the five-item scale, referring to mental work load, organization constraints on task completion and conflicting demands. Response choices were presented on a four-level Likert-type scale, ranging from one (“strongly disagree”) to four (“strongly agree”) and a sum score was calculated to measure job demands. An example item is: “My job requires that I work very fast”. Decision latitude was composed of the sum score of two subscales: “skill discretion” consisted of six items referring to the level of skill and creativity required on the job and “decision authority” was composed of three items concerning the possibilities for workers to make decisions about their work. Responses on these items ranged from one (“strongly disagree”) to four (“strongly agree”). An example item is: “My job allows me to take my own decisions”. Job strain was defined as the ratio of job demands over decision latitude.

#### Bullying

Bullying was questioned using nine items, based on the scale of Quine [44]. Three items refer to “isolation”, four items assessed the dimension “destabilization”, while the dimension “threat to personal standing” was measured using two items. Response options on all nine items were: “yes, absolutely”, “rather yes”, “rather no”, “absolutely not”. An example item of the “isolation” dimension is: “At my work, necessary information is withheld from me”. An example item of the “destabilization” dimension is: “My efforts at work are constant undervalued”. An example item of the “threat to personal standing” dimension is: “I am a victim of verbal and non-verbal threats”.

#### Sickness absence

The registered sickness absence data were obtained from the personnel administration departments of the participating companies during 12 months follow-up. In Belgium a medical certification for absences of more than one day is required, to benefit from guaranteed salary and medical insurance. Subsequently, the sickness absence registration is expected to be highly accurate. Complete sickness absence data could be gathered for 2876 participants; 107 were lost during follow-up. This drop-out was mainly due to resignation or dismissal, and not attributable to health-related reasons.

Former research investigating the relation between bullying and sickness absence spells of a certain duration considered sickness absence spells varying between 4 days and 6 weeks [12, 20, 22], revealing an inconsistency regarding the definition of sickness absence. Since earlier studies clearly demonstrated a relationship between bullying and depression and mental health problems [45], we hypothesized that bullying possibly harms the health of the worker, rather than it would solely reflect the coping behavior as an attempt to escape from the negative environment. Consequently, we decided to use a measure including long-term sickness absence spells, reflecting the health status of workers [46] and explicitly not to focus on absence frequency in terms of number of episodes (which is known to be more related with coping behavior). Since the time lag between exposure and outcome was only 12 months, it is not warranted to restrict the outcome to particularly long-term sickness absence spells of for instance 4 weeks or more. In this study, a long spell of sickness absence was defined as at least 15 consecutive calendar days of sickness absence during the follow-up period.

#### Covariates

The respondents were questioned about several socio-demographics, health behaviors, self-rated health, the occurrence of long-term sickness absence during the preceding year and neuroticism. The factors included as covariates were considered to be potential risk factors for sickness absence and could therefore act as confounders of the relation between job strain, bullying and sickness absence [47].

Socio-demographic control variables included age (continuous variable), gender (male/female), educational level and occupational group. Low educational level was defined as primary school and the first 3 years of secondary school level, medium education as secondary school level and high education as high school or university. Occupations were defined according to the International Standard Classification of Occupations [48] and grouped into executives, white collars and blue collars. Company was retained as a possible confounding variable, since important differences in work stressors and sickness absence are known to occur between companies.

Health behaviors comprised current smoking habits (yes/no), alcohol use and body mass index (BMI). Excessive alcohol consumption was defined as an average of more than three units per day for men and more than two units per day for women [49]. BMI was calculated as the self-reported body weight (in kg) divided by the square of the reported height (in m) and was entered as a continuous variable in the analyses. Self-rated health was evaluated by the following question: “How do you generally assess your health?”, with five response categories. The variable was dichotomized: very good or good versus average, bad or very bad. The respondents were also questioned if they had a long-term sickness absence (at least 15 consecutive days) episode during the preceding year (yes/no).

Finally, a measure to assess the personality factor neuroticism was included in the questionnaire. One of the main problems for the interpretation of causal relationships in stress research, is the effect of “third variables”, which possibly affect the stressors and the outcome by using the same method [50]. Since personality plays a role in the perception of job strain, bullying and the attitude towards sickness absence, we included a personality factor as confounding factor in the model. The personality theory is mostly dominated by the five-factor model [51]. Of these five factors, especially neuroticism, which is considered as a general tendency to experience a negative affect, such as fear, sadness, or anger is expected to be involved in the response to stressors [52]. Therefore, the model was adjusted for neuroticism. Neuroticism was measured, using a scale, derived from the NEO Five-Factor Personality Inventory, consisting of 12 items. Respondents were asked to rate on a five-point Likert-type scale (one = strongly disagree; five = strongly agree), the extent to which each statement corresponds to their perception of themselves.

### Statistical analysis

Structural equation modeling was performed with Mplus version 6 software [53]. The Weighted Least Squares Means and Variance adjusted (WLSMV) estimation method was used for binary dependent variables. Scaling of the latent variables was done indirectly by fixing the factor loading of the first observed item at one. Pairwise deletion was used for handling missing data with categorical outcomes, which resulted in an effective sample size of 2376 employees. A number of fit indices were considered to assess the fit of the proposed model to the empirical data [54]. The overall χ2 fit index is reported but is not used for drawing conclusions on model rejection, since it is known to be largely influenced by sample size, tending to over-reject models with large sample size. Moreover, the χ2 fit index is known to behave differently in case of application of the WLSMV estimation method and inflates with non-normal outcome data [54]. For the Root Mean Square Error of Approximation (RMSEA), a value < .06 was considered as a good fit, a value < .08 was considered as an acceptable fit and a value > .10 led to rejection of the model [55, 56]. For the Comparative Fit Index (CFI) and the Tucker–Lewis Index (TLI), a threshold value > .90 was considered as a good fit [57]. Standardized factor loadings > .50 were perceived as good, loadings > .40 indicated an acceptable correlation and those < .40 were perceived as low. Estimation of the mediation proportion was calculated according the formula for a model with one intermediate variable, which is the ratio of the parameter estimates of the indirect effect over the total effect [58].

Before specifying the hypothesized relations among the study variables, we estimated the measurement model for bullying, by conducting an exploratory factor analysis (EFA), followed by a confirmatory factor analysis (CFA). The measurement model for bullying was integrated in the full structural model, to test the mediation model (Fig. 1).

Job strain, defined as the ratio of job demands over decision latitude, was included as an observed variable in the structural model. This approach was selected in order to linearly model the balance between job demands and decision latitude which enables assessing the impact of a continuous measure of exposure. This method was preferred above the median split procedure to avoid losing information by dichotomizing scales. The Flemish version of the JCQ showed good reliability and validity in previous Belstress study samples [59]. Moreover, a preceding EFA in the present study, showed reasonable results for the expected three-factor solution (RMSEA = .083; CFI = .946; TLI = .906; χ2 = 1123.497, df = 52, *p* < .001), revealing the scales job demands and the two subscales (decision authority and skill discretion) of decision latitude. Factor loadings were acceptable, except for the items “conflicting demands” and “repetitive work”, which was in line with earlier research [2].

The 11 questionnaire based covariates, mentioned above, were treated as exogenous variables and predicted the main variables in the model (job strain, bullying and sickness absence). All covariates were observed items, except for neuroticism, which was measured by a 12-item scale. However, we included neuroticism also as an observed variable, since the neuroticism scale is a widely used and sufficiently validated instrument, which has been developed as a clear separate dimension within the five-factor personality model [51].

## Results

Table 1 (sample characteristics) demonstrates that the majority of the sample was white collar or executive and that only 20 % of the total study sample was lower educated. Mean age was 43,3 (+/− 6,74) years. About 18 % of the sample had at least one period of LTSA during follow-up.

**Table 1:** Descriptive socio-demographic, life style, health- and work-related variables in the total study sample

Variable	Total study sample (*n* = 2983)
Socio-demographic variables:	
Sex: *n* (%)	
Men	1372 (46)
Women	1611 (54)
Age (years): mean (SD)	43.3 (6.74)
Educational level: *n* (%)	
Low educated	617 (20.8)
Medium educated	1031 (34.7)
High educated	1323 (44.5)
Occupation: *n* (%)	
Executive	719 (25.2)
White collar	1826 (64.1)
Blue collar	305 (10.7)
Lifestyle variables:	
Body Mass Index (kg/m^2^): mean (SD)	25.1 (4.08)
Smoking: *n* (%)	816 (27.5)
Excessive alcohol use: *n* (%)	619 (21.1)
Health-related variables:	
Poor self-rated health: *n* (%)	943 (32.1)
Work- related variables and sickness absence figures:	
Job strain: mean (SD)	0.46 (0.12)
Range: 0.13–1.58	
Bullying: mean (SD)	13.7 (4.85)
Range: 9–36	
Long term sickness absence: *n* (%)	522 (18.2)
Self-reported long sickness absence during preceding year: *n* (%)	555 (21.9)
Personality	
Neuroticism: mean (SD)	30.8 (8.14)
Range: 12–60	

In Table 2, the intercorrelations (Pearson correlations) for all study variables and constructs are presented. All correlations between LTSA and other study variables were significant but small in effect size (*r* <.20).

**Table 2:** Means, standard deviations and (Pearson) correlations among study variables

Variables	M	SD	1	2	3	4	5	6	7	8	9	10	11	12	13
1. Age	43.3	6.74	1												
2. Gender			-.03	1											
3. Educational level			-.15***	.11***	1										
4. Occupation			-.01	.06**	-.49***	1									
5. Smoking			.05**	-.00	-.15***	.09***	1								
6. Alcohol consumption			.08***	-.08***	-.08***	.03	.10***	1							
7. Bmi	25.1	4.08	.14***	-.16***	-.17***	.07***	-.06**	-.00	1						
8. Self-rated health			.14***	.05**	-.11***	.11***	.14***	.06**	.16***	1					
9. Bullying	13.68	4.85	.03	-.05**	-.12***	.13***	.05**	.02	.08***	.21***	1				
10. Job strain	0.46	0.12	.00	.16***	-.04	.11***	.06**	-.06**	.03	.18***	.38***	1			
11. Long sickness absence			.08***	.07***	-.13***	.11***	.08***	.04*	.10***	.17***	.10***	.10***	1		
12. Previous sickness absence			.02	.05**	-.12***	.15***	.09***	.04*	.06**	.20***	.11***	.10***	.18***	1	
13. Neuroticism	30.80	8.14	.06**	.18***	-.04*	.12***	.07***	.06**	.00	.32***	.36***	.30***	.13***	.12**	1

Notes: *N* = 2983; *p < .05; **p < .01; ***p < .001

### Measurement model bullying

In a first step, the measurement model for bullying was checked with an EFA, which resulted in a three factor solution with an acceptable to good model fit (RMSEA = .070; CFI = .996; TLI = .987; χ2 = 188.464, df = 12, *p* <.001) and factor loadings > .50. This three-dimensional structure was in line with the expected structure of the original instrument: a factor “isolation” was derived, loading on two items (withholding information; ignoring); a “destabilization” factor, which loaded on five items (unreasonable refusal of applications for leave; shifting of goal posts; undervaluing of efforts; demoralization; removal of responsibility areas) and a “threat to personal standing” factor loading on two items (threats; inappropriate jokes).

A first-order CFA with three factors revealed high modification indices relating to covariance between residuals of some of the items. Based on these modification indices and on theoretical assumptions that some items may have common causes other than the latent factors of the proposed model, four covariances between error-terms were allowed for. Firstly, covariance between the error-terms of the “demoralization” item (dimension “destabilization”) and both the “threats” and “jokes” items (dimension “threat to personal standing”) were allowed for, since persistent and constant demoralization can also be considered as a threat to personal standing. Second, covariance between error-terms of the “refusal” item and the “shifting of goal posts” item were tolerated, which are in fact both behaviors typically occurring in a hierarchical situation, that pushes the victim in a passive, uncontrollable situation. Finally, also covariance between error-terms of items “ignoring” (dimension “isolation” and “jokes” (dimension “threat to personal standing”) were allowed for: ignoring in an extreme form can be perceived as a personal threat and mockery. This first-order CFA with three factors (RMSEA = .079; CFI = .989; TLI = .983; χ2 = 471.460, df = 24, *p* < .001), also demonstrated high correlations between the factors (> .76).

A second-order CFA with the three factors at the first level and one overall factor at the second level, demonstrated a good model fit (RMSEA = .048; CFI = .996; TLI = .994; χ2 = 165.134, df = 21, *p* <.001) with factor loadings > .70. Small negative residual variance for the first-order factor “destabilization” could be observed: the correlation with the bullying factor was 1.001, indicating that the first-order factor “destabilization” is a major indicator of the second-order factor “bullying”. Therefore, the residual variance of the “destabilization” factor was fixed at zero [53]. The measurement model was overidentified, which allows interpreting the fit indices.

This measurement model was retained as final model, to integrate in the full structural model.

### Final structural model

**Fig. 2:**
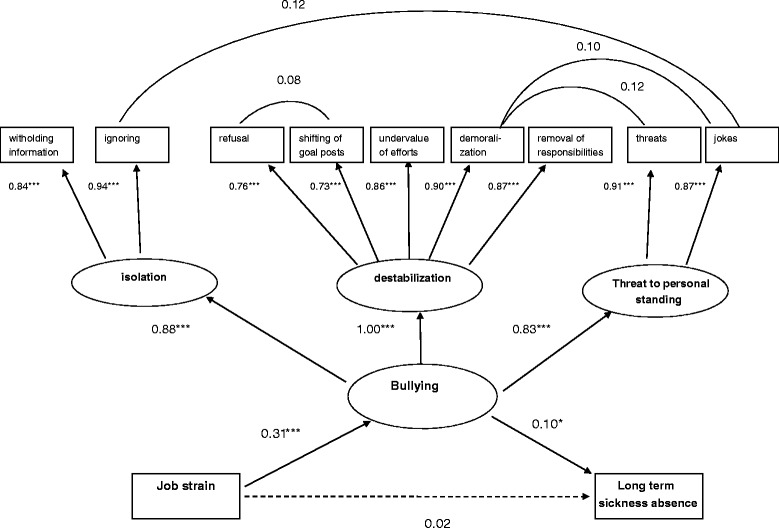
Standardizes parameter estimates for the final structural model. Model fit: RMSEA = 0.034; CFI = 0.998; TLI =0.983; χ^2^ = 476.086, df = 125, *p* < .001. *Dotted lines*: not significant; ***p-value < 0.001; **p-value < 0.01; *p-value < 0.05

After establishing a measurement model for bullying, the proposed hypotheses were examined. In Fig. 2, the standardized path coefficients are displayed in the final structural model, which demonstrated adequate goodness of fit measures (RMSEA = .034; CFI = .998; TLI = .983; χ2 = 476.086, df = 125, *p* <.001). This model revealed that job strain was significantly related to the latent bullying factor, which confirms hypothesis 2 (assuming high levels of job strain are positively associated with high levels of bullying). In line with hypothesis 1 (which assumes that high levels of bullying are positively associated with long-term sickness absence during follow-up), bullying was significantly associated with LTSA, while no direct association between job strain and sickness absence was demonstrated.

In Table 3, the direct relation of job strain and indirect relation of job strain through bullying with LTSA are presented. From this table, a significant indirect association can be derived, suggesting that bullying is an intermediate variable between job strain and LTSA, which therefore supports our third hypothesis (postulating that job strain is indirectly associated with LTSA through bullying). Calculation of the mediation proportion demonstrated that about 60 % of the relation between job strain and LTSA could be explained by the indirect association through bullying.

**Table 3:** Direct and indirect effects of job strain on sickness absence, using structural equation modeling

	Standardized parameter (S.E)	*p*-value
Direct effect	0.02 (0.27)	0.59
Indirect effect through bullying	0.03 (0.12)	0.02*

Notes: *SE* standard error.; * <.05; n=2376

Table 4 displays the standardized path coefficients from the covariates to the main variables in the model.

**Table 4:** Standardized path coefficients for covariates in relation to main variables

	Main variables		
Covariates	Job strain	Bullying	Long-term sickness absence
Gender	.103***	-.163***	.107**
Age	-.032	-.013	.060
Educational level	.000	-.029	-.066
Occupational group	.052*	.046	.074**
Company	.004	-.069**	-.129***
Smoking	.029	-.003	.052
Alcohol consumption	-.062**	.007	-.018
BMI	.043*	.024	.089**
General self-rated health	.057**	.074**	.091**
Previous long term sickness absence	.036	.024	.148***
Neuroticism	.253***	.314***	.067

Notes: *p <.05; **p <.01; ***p <.001; *N* = 2376

### Supplementary analyses

An alternative analysis was conducted, to examine the reversed pathway. Theoretically, it could be assumed that bullying leads into a deterioration of the work environment, with increased job strain, which would subsequently lead to sickness absence. However, the results of this analysis only showed a direct significant association between bullying and sickness absence (*p* < .05), while no evidence for and indirect relation (*p* > .05) through job strain was observed. Consequently, no support was found for the reversed pathway.

## Discussion

The main purpose of this study was to analyze a mediation model in which the indirect association of job strain with bullying on LTSA was estimated. We believe that this study contributes to the existing literature on job stress and bullying, since it enhances scientific understanding of the complex interplay between both stressors, using new statistical methods in the field of causal inference. An objective, prospective outcome measure was opted for and alternative analyses were conducted to compare their fit to the data.

Mediation analysis, using structural equation modeling, was applied to estimate the direct and indirect associations of job strain with sickness absence. An additional analysis (results not shown) was conducted to exclude the possibility of moderation: no significant interaction effect between bullying and job strain in relation to LTSA was observed. Overall, our results thus suggest that exposure to job strain creates circumstances in which a worker may likely become a target of bullying, which in turn is related to sickness absence. Bullying may be an intermediate pathway through which job strain is related to increased sickness absence. This finding is generally in line with the “work environment hypothesis” of bullying [11, 28], which is now followed by most investigators in bullying research. This situational interpretation emphasizes the importance of the organizational work factors, such as bad job content, in the origin of bullying. Several authors indeed demonstrated that the occurrence of bullying was significantly associated with a number of environmental work characteristics [11, 25, 28–30]. The work of Ballien further enhanced insights in this field, by explaining bullying in terms of the most leading job stress model of Karasek as a conceptual framework. Generally, job demands increase the probability of being a target of bullying, while high control protect against being bullied [35, 36]. This was also suggested by Takaki et al. demonstrating bullying as a mediator in the relation between job strain and sleeping problems and depression [41]. Accordingly, our research extends these findings by underscoring bullying as an intermediate variable in the well-established relation between job strain and sickness absence. Although the effect size of the observed indirect effect is small, the importance of our results should not be neglected. These findings have to be seen in the light of former research revealing that job strain accounts for a rather restricted amount of the variance in sickness absence [60], which is essentially defined by multifactorial causes. Moreover, the psychosocial work climate has the potential of being ameliorated through preventive measures on a collective basis, which may be, although reaching a minor effect, possibly more efficient than implementing worker specific and more health oriented preventive measures.

A second important result is that we could not find support for the opposite pathway: job strain was not an intermediate variable in the relation between bullying and sickness absence. This result, additionally adding evidence to our third hypothesis, is in line with the findings of Ballien et al. [36], who also found no support for reversed causation in their cross-lagged study. Generally, these results indicate that bullying did not have a harmful effect on the work environment and therefore contradict alternative stress frameworks, such as the “model of conservations of resources”, which is based on the supposition that people strive to retain their resources and that what is threatening to them means a potential loss of these resources [61].

Furthermore, our findings underline the value of one of the most influential and dominant models in job stress research and add evidence for the explanatory use of the JDC-model in the origin of bullying. Also Ballien demonstrated that this framework is valuable when investigating antecedents of bullying [36, 37].

Finally, our study adds evidence to the scarce literature revealing bullying as a predictor of sickness absence, which was until now only demonstrated in study samples, mainly consisting of health care workers [20–23].

### Methodological considerations

This study has some drawbacks that need to be mentioned. The main limitation is that both job strain and bullying measures are based on cross-sectional self-reports. Several precautionary measures were taken to reduce common method bias in our results: confidentiality was guaranteed to lower social desirable answers, sickness absence measures were based on objective registrations and the relations were adjusted for several possible “third variables”, including a measure for negative affectivity [62]. Because of the cross-sectional nature of the questionnaire assessment, causality of the relations cannot be established. Even if additional analysis exploring the reversed pathway showed no indirect association of bullying through job strain, which is moreover in line with the theoretical background of the work environment hypothesis, our study design does not permit concluding that job strain causally effects LTSA through bullying. In order to counteract the limitation that no statistical control could be conducted for prior measures of the main variables in the mediation model, substantive control of confounding was taken care of. Particularly baseline self-reported LTSA and negative affectivity are very likely to correlate with prior exposure to job strain, bullying and LTSA. The second limitation is the rather low response rate, which possibly leads to a selection bias in the study sample. Although no important differences in age and gender were revealed, we were not able to investigate if sickness absence levels differed between non-respondents and respondents. Third, it should be noted that participants of the Belstress III study were not recruited from a representative sample of the Belgian working population. Therefore, caution should be made in generalization of the results. Nevertheless, representativeness is less crucial than variation in exposure in analytical studies like this one, where possible relationships are examined [63]. A fourth limitation, is the use of the bullying questionnaire based on the Quine inventory. This scale has rather limited application until now and has the disadvantage that a recall period is lacking. However, using this scale has also an important strength: the results are based on multiple items per dimension, which enables assessment of the psychometric quality This scale has however some advantages: the results are based on multiple items per dimension, which enables assessment of the psychometric quality of the inventory. With CFA it was possible to recognize the different latent factors, proposed by the author, which additionally support the validity of this questionnaire. Another issue worth noting, is the rather short follow-up period of one year. Future research has to establish the ideal time frame to investigate the full effect of job strain on bullying and sickness absence. Finally, although the variable ‘company’ was entered as a covariate in the model, this approach does not allow accounting completely for the clustered design. Several additional analyses were performed such as repeating the main regression analysis from the mediation model using a generalized linear mixed model approach, and repeating structural equation models stratified for company. These additional analyses overall confirmed the findings and conclusions presented in this paper.

Besides these limitations, some particular strengths have to be mentioned. There is the use of the structural equation approach to assess the mediation model, which is argued to be superior to the more conventional Baron and Kenny’s method, to establish mediation, since a simultaneous estimate is made instead of assuming three independent equations [42]. With respect to this specific approach, it should be noted that assumptions required for a reliable estimation of the parameters were fulfilled. Firstly, the sample size, which is advised to be at least 10 times the number of freely estimated parameters in the final, structural model, was sufficiently large. Second, the measurements for the mediator and the outcome can be assumed to be largely free of measurement error. The outcome (LTSA) was based on objective, prospectively registered sickness absence measurements, which are obviously more reliable than self-reported figures. For bullying, the measurement model shows a factorial structure corresponding to the proposed model and demonstrates good fit with the data, which underlines the construct validity of the bullying measure. Third, the possibility of unmeasured common causes of the main variables has to be excluded. Therefore, multiple possible confounding factors were integrated in the final structural model, including baseline LTSA, self-rated health and a personality factor assessing negative affectivity. Finally, the world wide used JDC-model was applied to assess job stress, which is a reliable theoretically-driven measure.

### Recommendations for further research

Although this study increases the insight in some important processes between job stress and sickness absence, many aspects in this area remain unclear. Therefore, several recommendations for further research can be made. A first issue relates to investigating whether sickness absence in bullying victims represents real ill-health (required to recover from the illness) or rather a coping behavior to escape from the adverse work environment. Additionally, this study only focused on actual sickness absence behavior as an outcome and therefore did not capture the complete attendance dynamic, since no presenteeism figures were included in the analysis. It is recommended to extend sickness absence figures with presenteeism, which will lead to more insight into the attendance dynamic as a behavioral decision process in response to the perception of several job stressors. A second aspect that needs further study, is the precise physiological mechanism through which bullying exerts its ill making effect. Third, also other job characteristics, such as job insecurity, cognitive and emotional demands should be integrated in a conceptual model to further elaborate the role of bullying in the effect of the work environment on sickness absence. Fourth, besides the interplay between bulling and specific features related to the work content, also the particular role of social support should be subject of study. Finally, studies with measurement of both independent, mediator and dependent variables on multiple time occasions, would allow getting more insight in the complex causal relationship between several stressors and the outcome.

### Practical implications

The main findings of this study yield some important implications for management strategies reducing sickness absence due to bullying. While former research underscored the importance of conflict management strategies in the prevention of bullying [64], this study also highlights that remodeling jobs to reduce job strain may be important. Our work reveals that using the JDC-model as a framework may be appropriate to prevent bullying and sickness absence. Reducing job strain (by lowering job demands and/or increasing control) may prevent bullying behavior on the workplace, which would have beneficial effects on the sickness absence figures.

## Conclusions

To summarize, we believe that our study offers a valuable contribution to the existing literature by establishing the important role of bullying in the relation between work characteristics and sickness absence. The results generally extend the widely accepted work environment hypothesis of bullying, by suggesting the intermediate pathway of bullying in the relation between job strain and LTSA.

## Abbreviations

BMI: Body mass index
CFA: Confirmatory factor analysis
CFI: Comparative fit index
EFA: Exploratory factor analysis
JCQ: Job content questionnaire
JDC- model: Job-Demand-Control-model
LTSA: Long-term sickness absence
RMSEA: Root mean square error of approximation
TLI: Tucker–Lewis index
WLSMV: Weighted least squares means and variance
